# Transgender and Non-binary Swimming in the UK: Indoor Public Pool Spaces and Un/Safety

**DOI:** 10.3389/fsoc.2020.00064

**Published:** 2020-09-03

**Authors:** Jayne Caudwell

**Affiliations:** Department of Social Sciences and Social Work, Bournemouth University, Poole, United Kingdom

**Keywords:** transgender, non-binary, inequality, swimming, in-door blue space

## Abstract

This paper draws from the findings of research that was initiated as a consequence of previous research activities related to University-LGBT community physical activity projects (2012–2018). Specifically, the research underpinning this paper centers transgender and non-binary experiences of recreational swimming and aquatic activity (2017–2020). To date, the research has received small amounts of funding from four sources and resulted in two public engagement activities (two art exhibitions). The findings that inform the discussion are taken from nine semi-structured interviews, three focus groups including a professionally drawn illustration of two of these focus groups, and sixty-three research participant's “drawings” as well as informal conversations with eight stakeholders. The findings concern transgender and non-binary people's feelings of un/safety in the public spaces of an indoor swimming pool and the accompanying display of their embodied self. These two elements of un/safety—spatiality and embodiment—are critically discussed in relation to physical activity and in/equality. In this way, the work contributes to sustained University-LGBT community links and provides possibility for evidenced-based intervention to address inequality.

## Introduction

Swimming in the United Kingdom has a social history contingent upon traditional notions of class, gender, ethnicity and empire, and health (Love, 2008). Indoor public pools were first built— and some remain in UK cities—between 1880 and 1920 as a form of Victorian public service provision (McLauchlan, 2017). The contemporary provision of indoor pools has been viewed as municipal with community, health, and well-being benefits (Moffatt, 2017), and as social welfare curtailed by UK Conservative Government policy such as Compulsory Competitive Tendering (CCT) and Austerity (Parnell et al., 2015).

Swimming as a physical activity engages participants at many levels including active leisure and recreation as well as elite performance and competition. In the UK, swimming has one governing body, but a multitude of commercial providers manage and staff public pool facilities. At the elite level swimming is a highly disciplined sport not without its issues, for example abuse of athletes (McMahon and Dinan Thompson, 2011; McMahon and Penney, 2012). At all levels, swimming is regarded as an activity that involves high surveillance, especially of the body (Lang, 2010), within confined space (Rinehart, 1998; Ward, 2017).

The public spaces and the embodied activity of swimming make it a complex form of physical activity. Despite these circumstances swimming remains popular in England; it was ranked sixth in the recent Active Lives Adult Survey (Sport England, 2019), with open water/outdoor swimming on the increase (Sport England, 2018). Research with women (Throsby, 2013) and older adults (Phoenix and Orr, 2014) demonstrates that people derive pleasure from swimming, and yet, swimming, and indoor public pools are often unwelcoming to transgender and non-binary people and communities (LEAP Sport Scotland, 2015; Elling-Machartzki, 2017; Jones et al., 2017b; López-Cañada et al., 2019). Related to recreational open water swimming, the publicly aired debate over whether transgender women can access London's Hampstead Heath women-only pond, signals how transphobia impacts opportunities to participate (Topping, 2018; Marsh, 2019). Additionally, the debate highlights how specific feminist perspectives can underpin transphobic response. Briefly, it is the notion that trans women threaten the category woman, the advocacy of women's space, the categories of sportswomen and women's sport. Some of this debate exists within the elite echelons of swimming and has had an impact on the transgender and non-binary community through compounding hostile and abusive treatment of people, especially on social media.

The emergence of exclusive transgender and non-binary swimming groups in UK cities might be viewed as the result of the belligerence embedded within transphobia. Examples of groups that have used public pools include, TAGS in London (see https://aeon.co/videos/freedom-power-and-water-turning-the-community-pool-into-a-transgender-safe-space), Seahorses in Glasgow, Marlin in Manchester, and City Council provision in Brighton and Hove (see https://www.brighton-hove.gov.uk/event/trans-friendly-weekly-swim). In this paper, I focus on a Bournemouth-based social group and its members that attended once-a-month transgender and non-binary swimming sessions during 2017–2019. Although there is evidence of increasing sport studies research with, and for, transgender communities and individuals (Travers, 2018), to date our knowledge of the context of swimming remains underdeveloped.

## Inequality: Conceptual Framing

Recent research-based literature demonstrates, through systematic review (Jones et al., 2017a) and meta-synthesis (Pérez-Samaniego et al., 2019), that there exists evidence of transgender people's negative experiences of physical activity, sport and competitive sport. For example, Jones et al. (2017a) found evidence of structural barriers to participation and a lack of inclusion through their critical scrutiny of sport policies (*n* = 31), and their review of existing academic research findings (*n* = 8). Pérez-Samaniego et al. (2019) provide valuable focus on existing qualitative studies (*n* = 12), highlighting shared findings of exclusionary processes such as abjection (Travers and Deri, 2011; Lucas-Carr and Krane, 2012), use of language (Semerjian and Cohen, 2006; Sykes, 2009), design of space and facilities (Lewis and Johnson, 2011; Van Ingen, 2011; Oakleaf and Richmond, 2017).

Both papers (Jones et al., 2017a; Pérez-Samaniego et al., 2019) suggest research commenced in 2006, and identify USA, UK, Canada, New Zealand, Netherlands, Northern Ireland, and Spain as the geographical contexts for the studies. This type of tracing of the research trajectory of transgender physical activity and sport participation is vital in informing future research developments. However, we cannot ignore that the existing lens is mostly on cultures pertaining to the west and global north. As such, it is easy to conclude that white transgender experiences currently dominate our knowledge; it is essential that we recognize this and make subsequent efforts to change the bias.

Recent studies (since 2015) demonstrate a number of items that cause exclusion of transgender participants from physical activity and sport. For example, changing/locker rooms, school sport, and public spaces (Hargie et al., 2017); how transgender people are imagined—by Spanish University sport science students—through tropes of abjection and alterity (Pérez-Samaniego et al., 2016); the binary arrangement of sport within UK University environment and policy (Phipps, 2019); both internal and external barriers and facilitators for young transgender adults (Jones et al., 2017b); embodiment, fear, transitioning, social support, physical education, and how space is regulated (López-Cañada et al., 2019); the body, pre- and post-transition, stigma and pride (Elling-Machartzki, 2017); and identity, participation, competition, physical embodied change, and coming out (Klein et al., 2018, 2019). Implicit to most existing studies is the element of safety and feeling safe.

These recent research-based studies as well as the previous studies highlighted by Jones et al. (2017a) and Pérez-Samaniego et al. (2019) have theorized findings through critique of: current policy and provision; past and present pedagogy and ethics of care; the obdurate sex-gender binary arrangement of sport; queer theory, transgender theory, feminism, queer-feminism, and transfeminism. Klein et al. (2018, 2019) are explicit in their transfeminist framing. This approach—transfeminism—is significant, given the broader debates within feminism and transgender studies (cf. Bettcher and Stryker, 2016). For instance, Gender Critical Feminists and Trans Exclusive Radical Feminists (TERFs) call for the exclusion of trans women from women-only communities, spaces, and places. Here, exclusion is based on largely ideological and philosophical arguments regarding “womanhood” and defining “woman.” Transfeminism enables an examination of exclusionary practices in view of transphobia. Moreover, as Bettcher (2017) posits, transfeminism provides robust scholarship for transgender equality.

Trans feminism explicitly proceeds from the recognition of the intersections of sexist and transphobic oppressions. Although this would appear to centralize trans women, since trans men are also vulnerable to sexism, transphobia, and the interblending thereof, trans feminism would be ill-advised to exclude them from its purview (p. 2).

Ahmed (2017) makes an interesting observation, during her discussion of works by transfeminist activists Stryker (1994), Stone (2006), and Serano (2007), when she suggests that transfeminism shares a “militant spirit” with lesbian feminism because “of the insistence that crafting a life is a political work” (p. 227). Through her examination of the three texts, she argues that transfeminist politics illuminate:

… not only how the sex-gender system is coercive, how it restricts what and who can be, but how creativity comes from how we survive a system that we cannot dismantle by the force of our will alone (p. 227).

The fights for equality and the connections made between transfeminism and lesbian feminism appears within Griffin's (2012) discussions of sport, sex, and gender. Her chapter entitled: “Ain't I a Woman?” starts with the selective nature of some strands of feminism in their advocacy for particular women, and their rights. Borrowing from Sojourner Truth's address to white feminist abolitionists to include black women, Griffin shows how sporting practices—often based on suspicion—exclude some women. In a critical engagement with “the myth of the level playing field,” Griffin compares how lesbian athletes were/are deemed a threat to women's sport with how transgender and intersex athletes are considered through distrust.

Although lesbians may be viewed as women who look or act like men, some people view transgender and intersex women as actually *being* men, in most places making them ineligible to compete … (p. 106).

For transfeminists, it is the confines and coercive nature of the sex-gender binary structure, especially in physical activity and sport, which is problematic and in need of change. However, as Ahmed (2017) identifies it is often left to transgender and non-binary people to challenge the arrangement through “a politics of transgender rage,” and the generative re-appropriation of the “monster” trope (p. 227). This transfeminist activism, by transgender and non-binary people, is in spite of being one of the most marginalized groups of people (Currah, 2016).

Marginalization has a number of effects in terms of inequality, and research demonstrates that individuals that belong to the LGBT+ community experience higher levels of anxiety, depression, and suicidal feelings as a consequence of feeling isolated, and experiences of rejection, harassment, and bullying (Connolly et al., 2016; Public Health England, 2017). For example, a recent UK Government survey concluded that: “LGBT respondents are less satisfied with their life than the general UK population. Trans respondents had particularly low scores” (Government Equalities Office, 2018). As such, health inequalities have a significant impact on the lives of transgender people.

Physical activity, in particular swimming and aquatic activity, has positive mental and physical health benefits (Swim England, 2017); swimming and aquatic activity participation have the potential to address health inequalities. However, Pride Sports (2016) show that transphobic language and the acceptance of transphobic “banter” pervade physical activity and sport environments. As highlighted above through previous research, many transgender and non-binary people experience sport and physical activity as discriminatory, prejudiced and abusive.

Transgender and non-binary people face a set of inequalities when it comes to physical activity participation. These inequalities impact on participation rates. In 2014, Public Health England claimed that 42% of LGBT+ people met the required levels of physical activity for “good” health, compared with 59% of non-LGBT+ people. In 2016, Pride Sports suggest that 55% of the LGBT+ community are not sufficiently active to maintain “good” health. Moreover, National LGB&T Partnership (2015) found that “64% of LGBT people who identified as something other than male or female (e.g., genderfluid or genderqueer) were not active enough to maintain good health.”

As previous research and surveys have shown transgender and non-binary inequalities include structural and ideological conditions related to pre-set assumptions of who can participate, when and how. Compounding these structural and ideological inequalities are the unequal social relations of the spatial and the embodied, which are often overlooked. This paper focuses on the overlooked and the intricate operation of inequality for a group of transgender and non-binary people that participated in private-hire recreational swimming sessions.

## Methodology and Methods

### Ontologies of Transgender and Non-binary

Similar to the naming and labeling of class, race, ethnicity, and disability, taxonomies of gender and sexuality exist as social constructs produced through time, culture, politics, and power. Previously, I have critically discussed ontologies of LGBT (Caudwell, 2014) and ontologies of sexualities (Caudwell, 2015), making the point that there a number of different ways to explain the nature of gender and sexuality as well as the reality of being LGBT. However, there are examples, from the past, that seek to fix down set classifications, which are often based on creating a “science” of gender and sexuality. This approach continues when sport governing bodies depend on medicalized demarcation of the body, specifically the sexed-body.

In this paper, transgender and non-binary are terms that reflect self-definition by the research participants. As with the language of lesbian, gay, and bisexual, there is an element of identity politics and socio-cultural cachet attached to “transgender” and “non-binary.” Notwithstanding the currency of the idiom LGBT, and the value of the terms: transgender and non-binary, it is impossible to assume homogeneity and stability across and within. For these reasons as well as the rejection of purely medical models, this research adopts self-identification. The group viewed self-identification as normal practice; it was reflected through their standard introductory activity, at their Friday evening gatherings, in the form of a pronoun round. This is when individuals self-identified as she/her, he/him, they/them. The importance of self-selected pronouns is that it is affirming and inclusive, whereas mismatching pronouns is viewed as degrading and derogatory (Zimman, 2018).

### Methods

As a relatively long term and variously funded project the methods deployed range from the traditional qualitative methods of focus groups and one-to-one interviews to observation, professionally drawn illustrations, and research participants' drawings as well as informal conversations with stakeholders. The project started in 2017 and continued in to 2020. It developed as a consequence of existing University-LGBT community activities (cf. Caudwell and Spacey, 2018), and newly formed University-LGBT community relations. The findings that are discussed in this paper come from nine one-to-one interviews and three focus groups. The production of an accompanying illustration of two of these focus groups reflects a non-traditional form of research findings, which has important value in the dissemination of findings to both academic and non-academic communities (some of the this material appears in the findings sections of this paper). This phase of the research, which adopts traditional and non-traditional qualitative methods helped inform and was supported by observation of the private-hire swim sessions (*n* = 11), attendance at the group's once-a-fortnight group meetings (*n* = 9) and informal conversations with stakeholders (*n* = 8). The illustration and specific crops from the illustration as well as the participant's drawings (*n* = 63) underpinned public engagement with the project through two art exhibitions held at the start of 2020 and in line with the annual February LGBT History Month. Audience evaluations of the art exhibitions took place, but this material is not included in this paper. In short, the research methods that inform this paper are interconnected and on going.

The research project gained ethical approval through the usual procedure at Bournemouth University. Participant information sheets and participant agreement forms were used for the interviews, focus groups, observations, and drawings to ensure informed voluntary consent. The participant agreement form included the following item, which was ticked by all participants: “I give permission for members of the research team to have access to my anonymised responses. I understand that my name will not be linked with the research materials, and I will not be identified or identifiable in the outputs that result from the research.” Pseudonyms are used in this paper to ensure anonymity.

The interviews and focus groups adopted a traditional format that involved an interview guide and thematic analysis of transcripts. Thematic analysis was conducted by hand in order to seek out significant patterns in the qualitative findings. The process followed five steps, namely: familiarization of data, verbatim transcription (of interviews and focus groups), generation of codes/labels, categorization, and defining themes (Braun and Clarke, 2006). The stages of coding and categorization led to the development of two substantive themes—un/safety and embodiment. These themes were used to frame categorization of the professionally drawn illustration and the 63 “drawings” presented by members of the group.

The observations of swimming sessions and attendance at group sessions adopted a short-term ethnographic (Pink and Morgan, 2013) element of “hanging out” with the group, building trust, sharing information about the project, and gaining group approval for ideas such as the art exhibitions. Over the period of time, some members of the group moved away, and sometimes returned, and new people joined in. In this sense the group was transient, but there was a small core of regular attendees. At the same time as working with the group, it was important to explore what was possible in terms of sustaining the swimming sessions on completion of the funding. This meant informal conversations with stakeholders to gather information about potential local and national provision and policy.

### Reflexivity

It is always important for researchers to critically reflect on their own power, positionality, and privilege. This is especially the case when completing research projects with groups that are marginalized. The researcher-researched dynamic is inherent with power relations as feminist scholars have highlighted (Stanley and Wise, 1993; Denzin and Lincoln, 2002); not least when academics seek outputs based on the lives of “Others.” Qualitative researchers are encouraged to interrogate their role within this relation in order to claim research that is decent and that has integrity.

As a cisgender^1^, white, academic (i.e., middle-class), I have socio-cultural power and privilege in a number of arenas, including the physical activity of swimming. For example, I am given easy access to the public spaces of swimming pools. In terms of my embodied self, I do not normally face everyday, frequent and deliberate scrutiny, surveillance and suspicion. In this regard, my positionality and privilege contrasts with the research participants and must be acknowledged as such. I do not experience the inequalities—embedded in space and embodiment—that are experienced by the research participants, and I do not want to objectify the inequalities that are evident. However, I do want to make visible processes, practices, and cultures of inequality faced by members of the LGBT community.

Returning to Ahmed's (2017) points about transfeminism, as theory, politics and activism, transfeminism has potential for trans allies to contest the normative sex-gender system and concomitant transphobia. The battles for equality should not be limited to the transgender and non-binary community. From the onset, this research project sought to demonstrate unequal treatment and inequalities of opportunity relevant to the research participants.

## Findings: Swimming, Safety, and the Body

After most of the private-hire pool sessions (*n* = 11), participants were asked to “draw” how they felt before, during, and after being at, and in, the swimming pool. The results of this request varied from artistic drawings, simple drawings, a number of words on the page, and written passages. The following is an extract from a written piece completed by one of the group (post-swim session “drawing,” 17th August 2019):


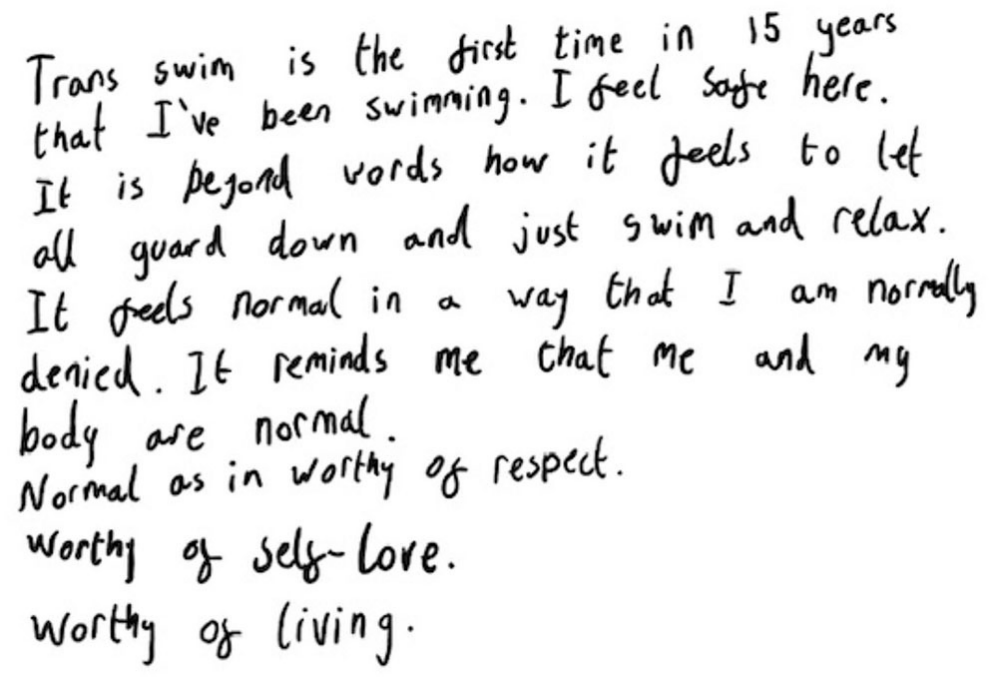


The theme of not swimming for a long period of time was shared by many of the research participants and mentioned in the one-to-one interviews:

Up until the first session [Trans Swim] I hadn't submerged in water, obviously I'd had baths, but like at the beach and swimming pools, for at least ten, twelve years I hadn't been swimming. (Joe, 22nd June 2018)

I didn't swim all these years. Probably nine years. (Sam, 10th May 2019).

The main reasons for ceasing to take part in swimming activities were contingent on being transgender or non-binary and linked with not feeling safe, and not feeling comfortable to display the body. The private-hire group swim sessions changed these conditions and made it easier to participate. For many of the group, it was the exclusive use of the indoor pool with familiar people that made the sessions a success, as depicted here in a post-swim session drawing (27th April 2019):


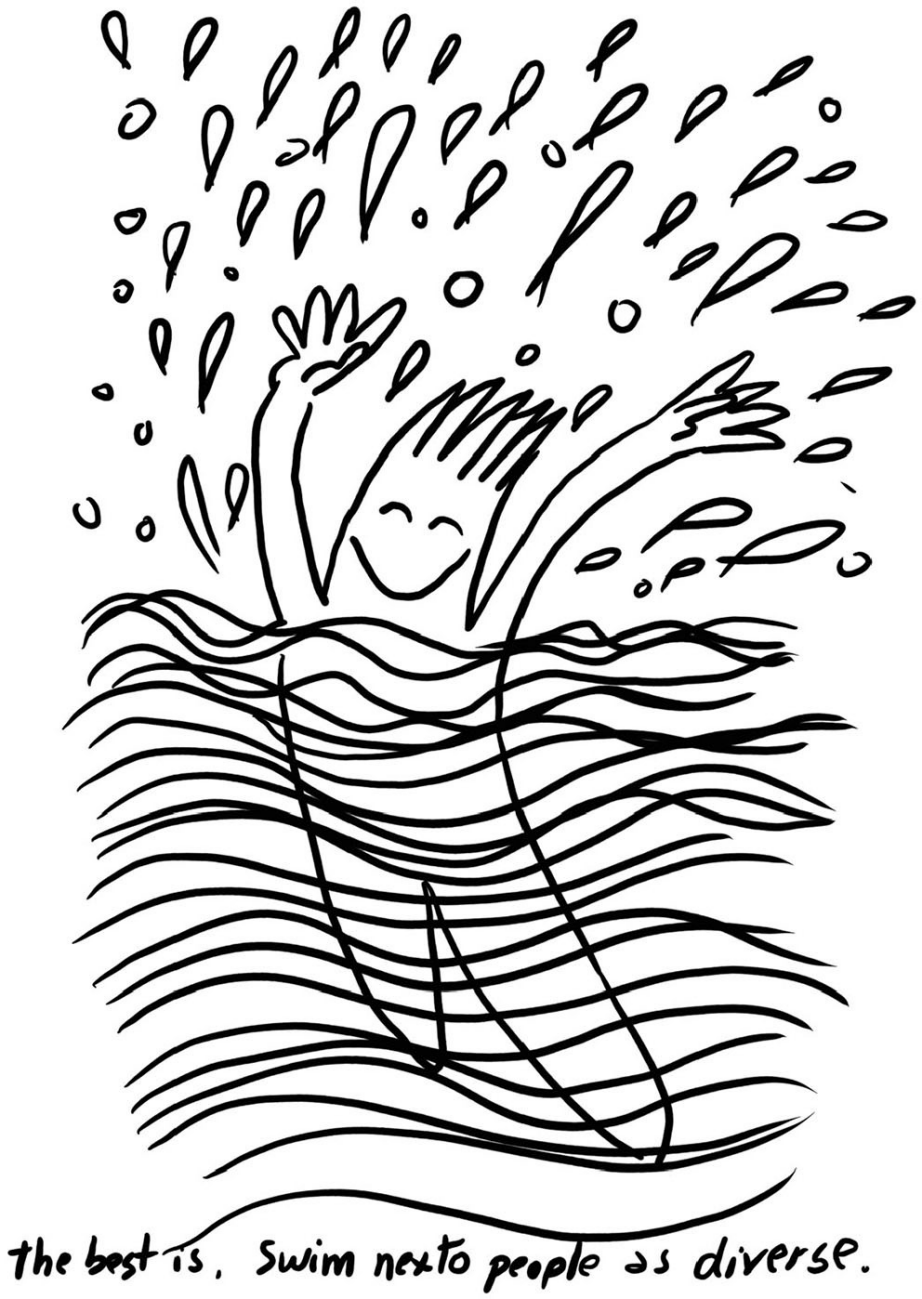


For some people, the sessions involved re-learning how to interact with the water: “When I started going, I couldn't swim at all. I hadn't been swimming in years and I got nervous being in the water. It took a lot for me to start putting my head under the water” (Fred, one-to-one interview, 10th May 2019). As such, not feeling safe and not feeling comfortable, in an embodied sense, were layered in terms of physical activity. For instance, apprehension was not only about entering public space and displaying the fleshy self, it was connected to the water-based physical activity.

For some of the group there were underlying fears that were not easy to identify, especially if portrayed via artwork, as is the case here (post-swim session drawing, 23rd November 2019):


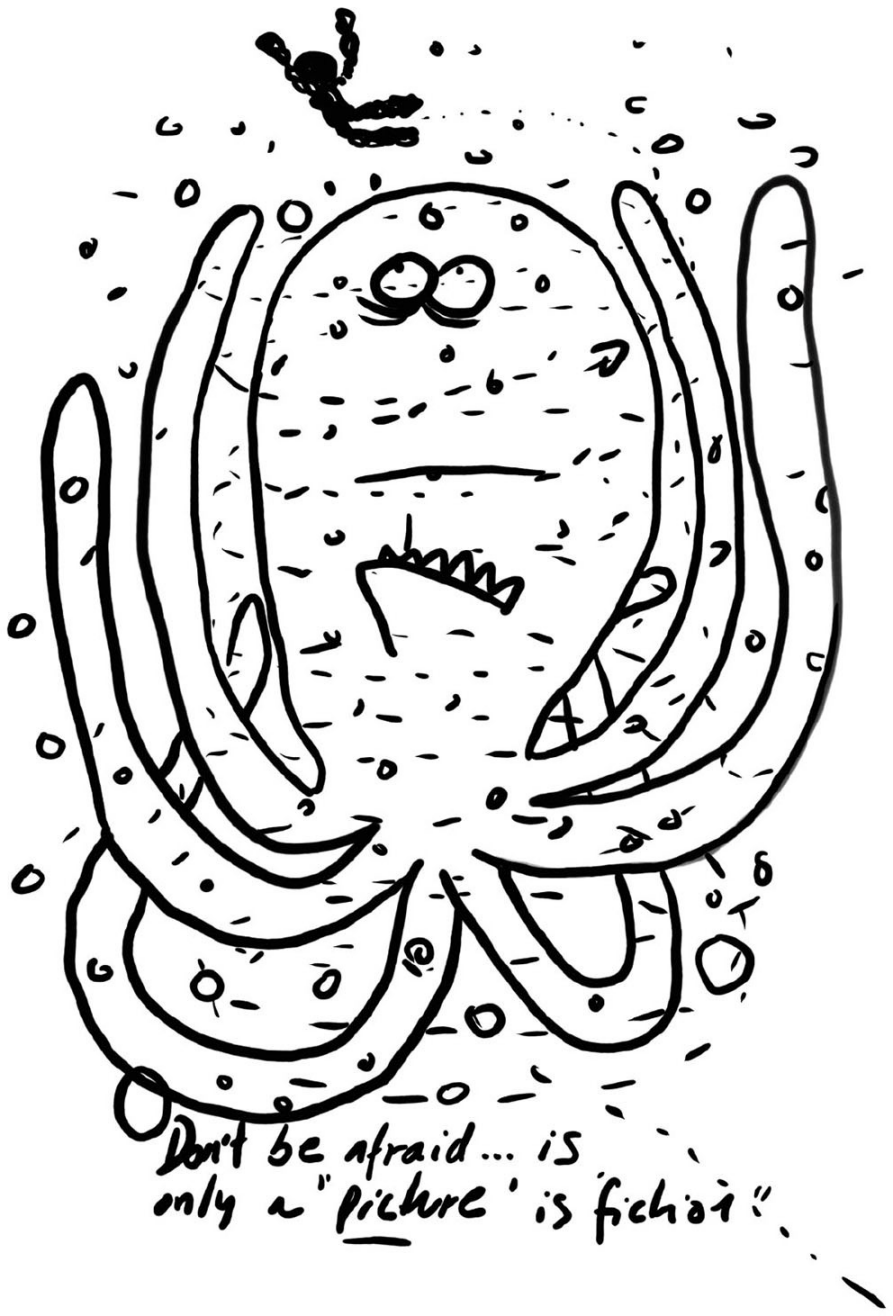


The one-to-one interviews and focus groups provided findings related, specifically, to safety and these findings help illuminate the caption in this picture: “Don't be afraid … is only a ‘picture' is ‘fiction'.”

### In/Equality and the Scales of Safe Space

Space, the spatial and spatiality have received critical scrutiny vis-à-vis gender and sexuality through a range of disciplinary lens including cultural geography (Bell and Valentine, 1995), feminism (Massey, 1994), and queer studies (Bell and Binnie, 2004), to name a few pioneers. Throughout, it is acknowledged that public space is imbued with power relations that operate to enable dominance, control and regulation. For example, Patel (2017) evidences racism, sexism and transphobia in bathroom (toilet) space in South Africa. Their work demonstrates high levels of visual, verbal and physical violence against transgender people of color. They refer to the processes as “violent cistems” in public toilet spaces, which includes the cisgender gaze that is used to govern transgender bodies, hostile exclusionary comments, and physical ejection. All three amount to “intensified policing” by cisgender people of what are viewed as “potentially contagious” (p. 8) transgender bodies.

Public visual and verbal violation of transgender bodies was talked about during the focus group research. For example, one research participant told of the impudent cisgender gaze and another shared a story about the effrontery they have faced:

… [i]n public, I am dressed and I am female presenting, the general public will look at me, look at my face and look down to my genitals and then look back at my face. … I don't get that from trans people. (Focus Group 3, 1st June 2019)

… if I am in public going to the toilets, I just get that fear; if I go on my own I am going to get picked on. I went to the toilet in Wetherspoons [UK pub], it wasn't too bad, but I was walking out and there was this old man: ‘are you in the wrong house?' I kind of laughed. (Focus Group 3, 1st June 2019).

Both of the above recounted incidents were acknowledged as familiar and shared experiences by other members of the focus group. The fear of visual and verbal surveillance in spaces and places meant that many of the research participants felt unsafe in public. As one of the interview participants put it: “Being trans you know you're not always going to be safe, there's going to be backlash, you're always going to be worried about things happening” (Robyn, 22nd June 2018). To go out into the public sphere is something that transgender and non-binary people have to consciously consider and plan for. For instance, this research participant felt safe only within specific conditions (company and time of day):

I go with my dad on walks because I know my dad is going to be there. I feel safe. We go quite late (…) and we have three dogs and they bark. It's just every time I am out in public, I feel like I am just waiting for someone to say something to me. (Focus Group 1, 9th November 2018).

Most forms of physical activity take place in public. The opportunity to go to a public indoor swimming pool as a group was highly valued by the participants. When asked, during one-to-one interviews, about the group's swimming session and exclusive use of the pool, comments ranged from the idea of “safety in numbers” and “amazing freedom” (Sam, 10th May 2019) to renewing the relationships with exercise and the self: “having a safe space for swimming has been the start of something amazing for me, it's got me back in love with fitness and myself” (Joe, 22nd June 2018). Previously the research participants experienced trepidation when swimming at a public pool: “I remember how terrified I was, I remember how afraid I was of being judged” (Aly, 22nd June 2018); “I never felt safe going alone” (Matt, 22nd June 2018); “I wouldn't feel safe enough to swim normally” (Sam, 10th May 2019).

Most of the swimming participants knew each other through the established social group and once-a-fortnight Friday evening gatherings. In these sessions, the group tended to focus on transgender and non-binary issues. The sessions were very supportive. In this way, a form of solidarity is built that was easily extended to the swim sessions. As one interviewee said:

“It's not easy to make friends with cis people because you can't really talk about the same stuff as you can with trans people. I find having the opportunity to make friends with other trans people is really valuable.” (Fred, 10th May 2019).

It is the collective understanding and appreciation of each other that enabled successful participation as well as helping some members to gain confidence:

I think having a space like [group name] is crucial to having swimming work so well because there's that trust built between us that everyone knows they're going to be comfortable … we know we are going to protect each other, we're going to be safe, and we made that safe space outside of our safe space. (Ean, one-to-one interview, 10th May 2019)

… the fact we go as a group and we have it closed off and we're all together and we all know each other, … helps bring people out of their shell in the pool. (Nic, one-to-one interview, 22nd June 2018).

During the focus group research, the group interactions reiterated these elements of support. For example, when research participants mentioned feelings of uncertainty, they would receive positive comments from other individuals in the group. Two crops from the professionally drawn illustration of the focus groups provide visual representation of this interaction:


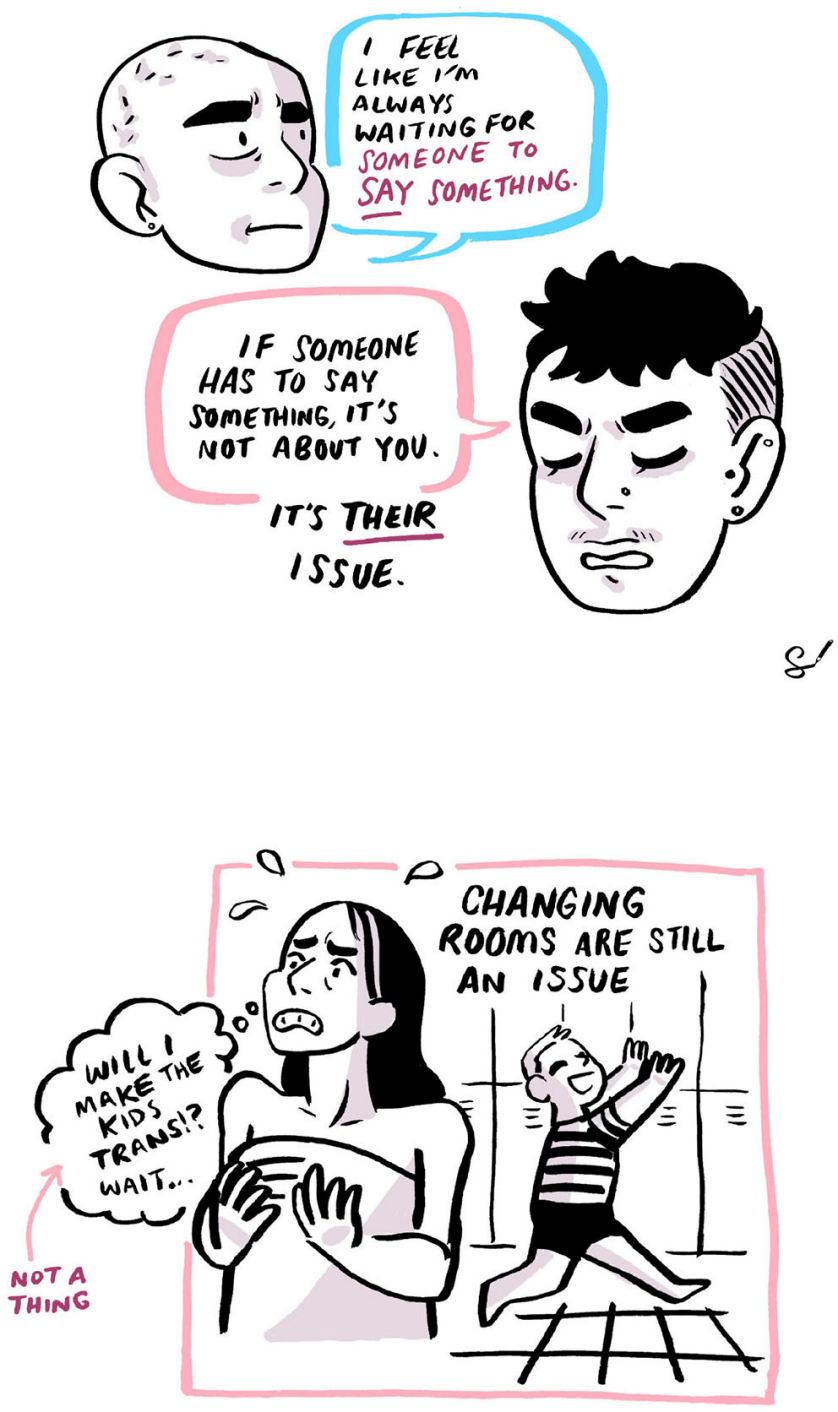


As with previous research (Caudwell, 2014; Hargie et al., 2017), changing rooms do present as a space within which transgender and non-binary people experience high levels of surveillance. The private-hire swim sessions took place once a month on a Saturday evening from 6 to 7 p.m. Saturday evenings were scheduled by the pool for 1-hour private hire and often this meant a children's pool party took place from 5 to 6 p.m. The changing room architecture conformed to village changing facilities, that is individual cubicles and not separate female and male changing rooms. The lack of binary changing facilities was one of the reasons why the group selected the venue. However, the changeover in usage between groups did remind some participants of previous anxiety and their transgender and non-binary presence in public space, as depicted above in the illustrations. During the discussion in the focus group one participant made the point:

I personally don't think about it [being looked at] once I am in the pool, because everything is gone then. I do when I am getting changed and I am like ‘OK, I can't hear kids anymore'. But, I'm not going “Whoa, I don't want the kids to see a trans person, because what if they become…” Just because you see something doesn't mean you're going to become that kind of thing. I watched Harry Potter as a child, but I didn't want to become a magician. (Focus Group 3, 1st June 2019).

For many of the research participants being in the water was the safest and most enjoyable space: “being able to go in the water and jump in; it's freedom. I love it” (Joe, one-to-one interview, 22nd June 2018). For those research participants that decided to write down how they felt before, during and after the pool session, common feelings for before were: anxious, nervous, shy, stressed, self-conscious, dysphoric, tense, awkward, restless, worried, and excited. Being in the water meant that many of the group felt free and liberated. After being in the pool, participants reported feeling: happy, content, calm, relaxed, confident, healthy, body confident, relieved, refreshed, peaceful, natural, energized, and motivated to do more exercise. In addition to these feelings, there is evidence of a collective pleasure:

I was in the water, the smaller pool, looking at the people in the bigger pool, and just seeing how happy everyone was. Everyone just looked happy and comfortable. To see everyone able to be themselves to be in their own skin and happy; it felt amazing. (Robyn, one-to-one interview, 22nd June 2018)

It was lovely to see my wife so happy too 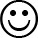 she had the best time! (post-swim session ‘drawing', 6th July 2019).

Not only was the water considered as a safe and pleasurable space, it was also a space of imaginative transgender and non-binary possibilities. For instance, this was written down after one of the swim sessions: TODAY, NOW, I AM A BEAUTIFUL DOLPHIN (6th October, 2018) and this was drawn (8th December, 2018):


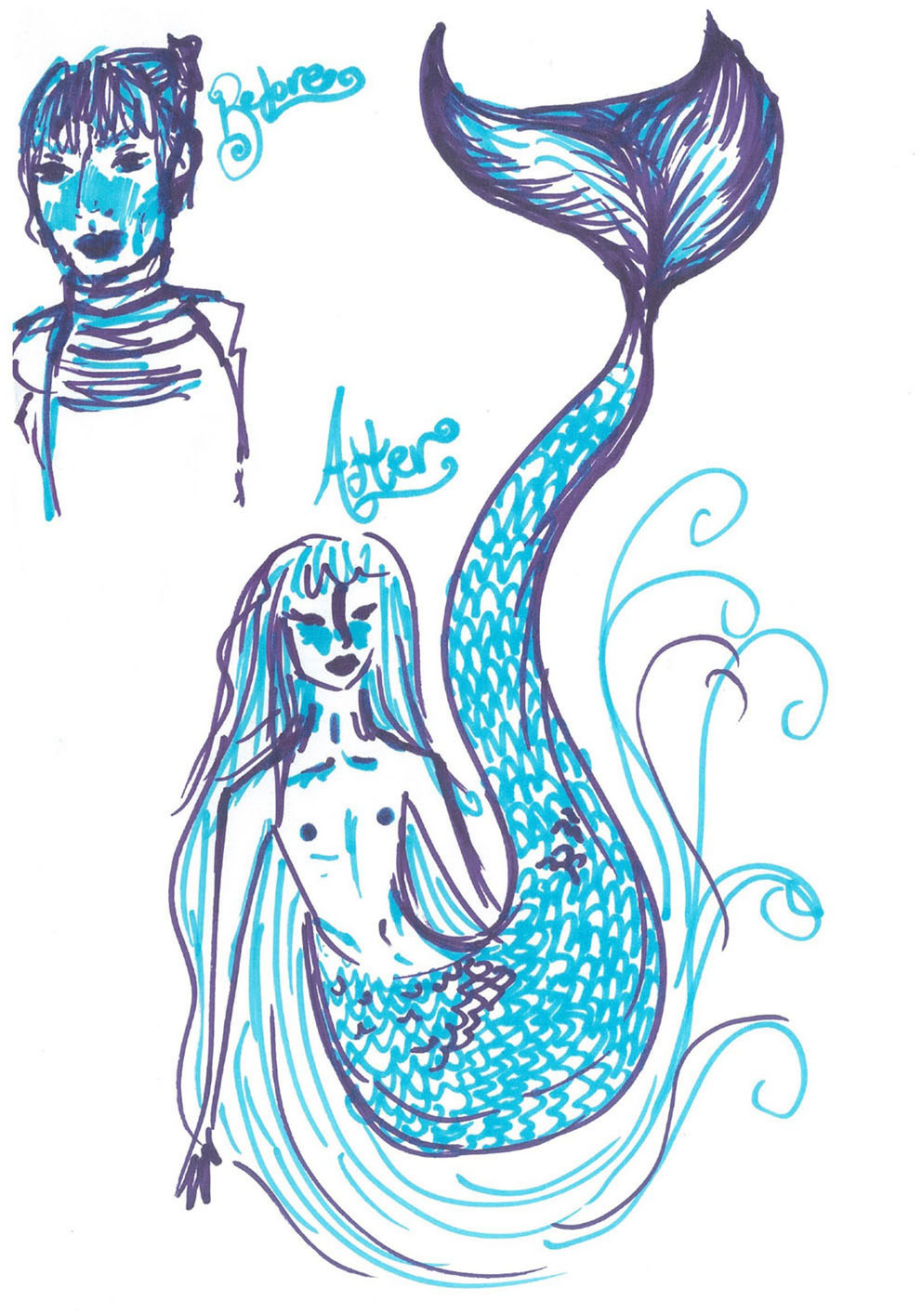


As Foley and Kistemann (2015) comprehensively outline, water, in its many forms, is known to connect with the health of individuals. Referring to health geography they show the significance of research of healthy blue space such as rivers, lakes and oceans, and accompanying landscapes, in understanding public health, health promotion, and individual well-being. Research of outdoor waterscapes reveals the therapeutic, restorative, and salutogenic value of blue space vis-à-vis swimming and immersion (Foley, 2015, 2017). The limited research of indoor blue space tends to highlight the history of spas and the explicit link to predominantly physical health (Foley, 2010). Despite an acknowledgment of the emotional, physical, and imaginative properties of being in blue space (Foley, 2015) and an appreciation of geographies of difference (Foley and Kistemann, 2015), we are unaware of how indoor watery safe space—i.e., public swimming pools—impact positively on transgender and non-binary people and communities. Foley and Kistemann (2015) recognize: “One characteristic of blue space is its capacity to embrace bodies of difference in ways that are gently enabling” (160). It is this immersion of physically active bodies of difference that I discuss next.

### In/Equality and Embodiments

In this paper, I argue that inequalities of opportunity and participation in physical activity, in particular swimming and aquatic activity, can be explained through an analysis of the spatial and embodiment. For the research participants in this research project, public space is viewed as mostly unsafe. This is because of how space is dominated, controlled, and regulated by people and practices that marginalize, even vilify, transgender, and non-binary individuals and communities. Public space is a significant component to configurations of inequality of access to physical activity for the research participants. Implicit to this research finding is transgender and non-binary embodiment in the context of indoor public swimming pools. For example, one research participant wrote after the swim session: “I get way too dysphoric to swim around cis people, so this [swims session] lets me swim” (27th April 2019).

Gender dysphoria is a complex term that is surrounded by medical discourse both physical (e.g., endocrinology and surgery) and psychological (e.g., psychiatry and mental health) as well as negative media commentary (Gregor et al., 2016). Often it is medical discourse that dominates responses to people that experience a mismatch in their sex-gender identity and sex-biological identity. Until 2013, the American Psychiatric Association classified gender dysphoria as gender identity disorder. Within the field of social work and social care advocacy more is now being done to work with individuals that experience the often-profound incongruences of gender dysphoria (Kameg and Nativio, 2018). Within sport and physical activity there is no evidence of a disciplinary or practitioner response to embodied gender dysphoria despite the embodied self being central to an individual's activity levels. As such, transgender and non-binary sport and physical activity participants can experience embodied inequalities that impact their levels of active engagement.

A number of research participants reported feeling embodied gender dysphoria prior to the swim sessions, especially surrounding what they were permitted to wear. Since the sessions were private hire, participants valued the ability to wear binders, t-shirts and shorts in the pool: “It felt good to not feel any pressure in what to wear … would never bind at a regular swim session” (post-swim session “drawing,” 19th January 2019). However, for one research participant they recorded that they felt “apprehensive about how I would feel as someone who has bigger breasts and is chubby wearing a binder & swim shorts.” The same participant also wrote about their feelings during and after the swim session, they exclaimed that it was: “Wonderful to be back in the water,” but after they felt: “Dysphoric having to get out of the water” (post-swim session “drawing,” 8th December 2018).

Not all participants were able to bind as they did not have a binder; they wore trunks only as is depicted here (post-swim session drawing, 6th July 2019):


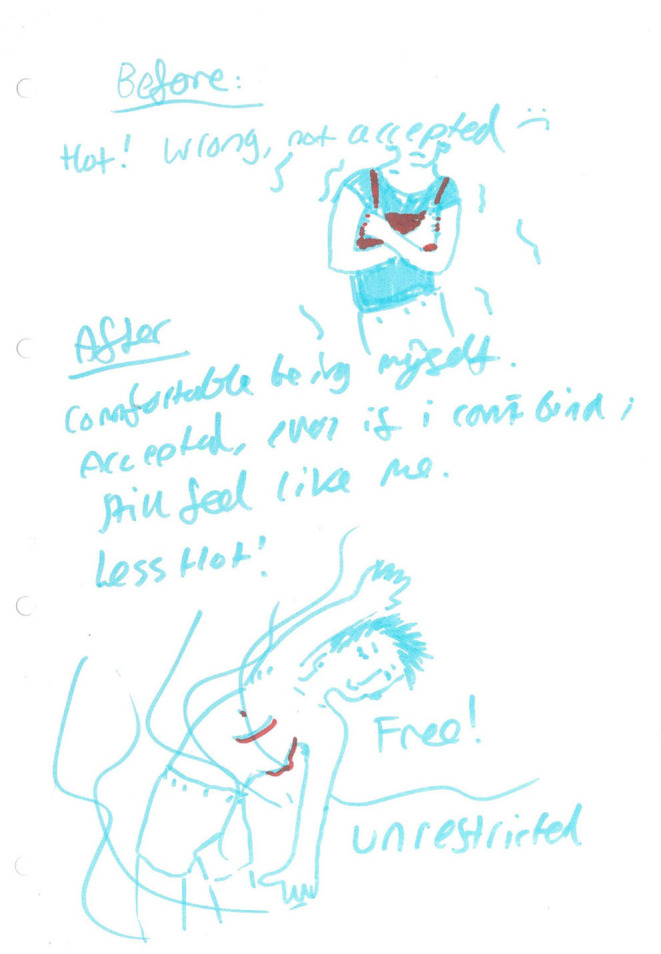


The emphasis on feeling: “[a]ccepted, even if I couldn't bind” was reiterated in comments referring to being judged: “During the swimming session I was having fun. I wasn't thinking about how others could be judging me because they weren't” (post-swim session ‘drawing', 21st September 2019); and “Swimming is great cos I'm free to relax and be myself with like-minded people who I know aren't going to judge me and won't be staring at my scares” (post-swim session ‘drawing', 23rd November 2019). For one participant, the experience of the swim session led them to express the idea: “Maybe I'll be able to go swimming with my mum again … if she can cope with the sight of me in a binder and trunks” (post-swim session ‘drawing', 6th July 2019).

Transgender and non-binary bodies can be transitioning bodies. This means that it is not only feelings of embodied gender dysphoria that impact the inclination to participate in physical activity, but processes of surgery. For one research participant, their transition was some time ago and they enjoyed the opportunity the swim sessions provided for positive display of the body: “I love to show my chest, because it's an ok chest” (Johan, one-to-on interview, 10th May 2019). Nic talked about similar positive feelings:

The fact that I didn't have to bind or wear a t-shirt was pretty cool. I know that other guys there [at swim sessions] have got scars as well. If it was just me and my regular friends outside the group, I'd feel very self-conscious because of the scars, but I didn't (one-to-one interview, 22nd June 2019).

In relation to the transitioning body, the private-hire swim sessions were regarded as supportive, positive and safe: “You know you are safe. No one is going to look—‘what's that?' on someone's chest” (Matt, one-to-one interview, 22nd June 2018). The swim sessions allowed participants to take part in physical activity at the same time as developing embodied confidence.

For me, I had to get used to people seeing my chest out; it's the safest environment for me in that situation. It started out that I wasn't comfortable as I wasn't used to it. It gave me the chance to get used to it and be comfortable… I am trying to encourage a friend, he's trans and has problems with his joints, so he can't do high impact stuff like running, but he can't go swimming anywhere else because of his chest, he doesn't feel comfortable. I'm trying to convince him to come. (Fred, one-to-one interview, 10th May 2019).

Exclusive use of the swimming pool by a transgender and non-binary group was immensely important for the participants, regardless of their transgender or non-binary identity. This exclusive use enabled physical activity: “Since top surgery, I still struggle with being misgendered so I find being in a trans safe group is the best way to exercise” (Ean, one-to-one interview, 10th May 2019).

During one of the focus groups, I pointed out that the group changes with different people attending. The response was: “That doesn't matter, as long as I feel safe and comfortable with the people” (Focus Group 2, 1st June 2019). The point was made that this extended to the poolside lifeguards: “They are watching us because they have to, because we are swimming. It's a different level of staring.” There was an incident during one of the sessions when one of the group (not wearing a t-shirt, not binding and pre-op) required assistance. During a one-to-one interview, Johan described the situation:

… the [life]guard had to get in [the pool] as they were having, not a fit, but something like that. They [lifeguard] were amazing, they just jumped in, held their head up for them. So in a way that makes you feel safer because you know they are doing their job (10th May 2019).

When someone mentioned the same incident during the focus group research there was expressed consensus with the comment: “I think the venue is quite safe.” The notion of safety discussed appeared to move beyond the initial point that the lifeguard on duty will take care of all swimmers' bodies and prevent them from drowning. This shift to a general sense of feeling safe is interesting because the stakeholder research, including informal conversations with two national governing bodies and the duty manager of the facility, indicated that indoor swimming pool staff do not normally receive formal training regarding transgender and non-binary physical activity inclusion. As such there is no informed knowledge of embodied issues such as dysphoria and use of language such as pronouns.

During the stakeholder research, it was evident from the informal conversations that people working for: indoor swimming pool facilities, local swimming provision, and national governance of sport were open, and willing, to support transgender and non-binary equality and inclusion. Researcher observation of the private-hire swim sessions recorded that the majority of responses from staff (receptionists, lifeguards, duty managers) were positive toward to the group and individuals. These responses are in the context of regular private-hire, exclusive use and the absence of a general public response, which can be transphobic and trans hostile.

There was one occasion when a lifeguard denied the group the use of inflatable objects in the pool. This was out of turn with previous practice. After the group made a complaint, the decision was reversed at the time by the manager on duty and later formally confirmed as incorrect via e-mail correspondence. Some of the group felt this one-off denial was based on a conscious move to have power and control over the group. After the swim session one participant drew a picture; it deploys humor as a way to ridicule the authority of the staff member (post-swim session drawing, 19th January 2019):


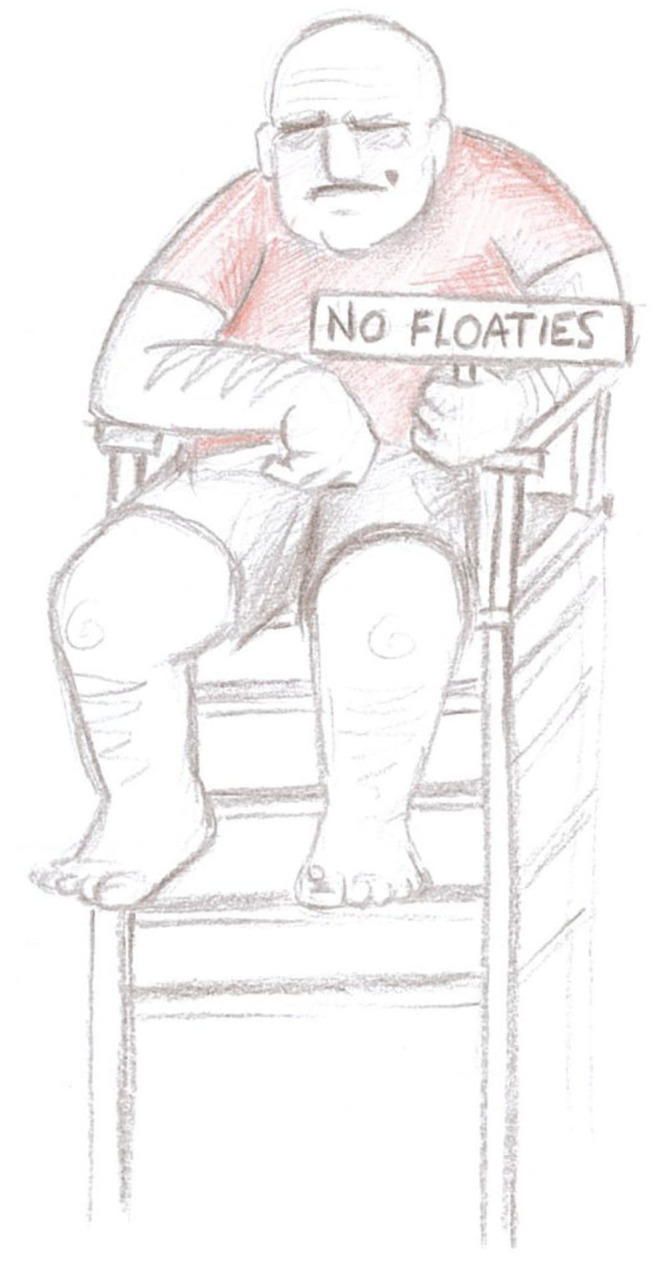


As trans feminists advocate, trans allies can take on the struggle for transgender and non-binary equality. There is some preliminary evidence from the research with stakeholders that they are keen to include transgender and non-binary participants. That said, we know that there exist a myriad of initiatives and policy concerned with inclusion and equality in physical activity and sport (Cunningham, 2019), but success of these initiatives and policy depend on enactment (Jeanes et al., 2019). For example, and specific to LGBT inclusion, implementation has received critical evaluation with researchers concluding that they are often not effective and can be described as “non-performing inclusion” (Bury, 2015) and/or organizations feel they “already do enough around equality and inclusion” (Phipps, 2020).

Despite embodiment being central to physical activity participation there is little evidence in this research project—to date—that stakeholders explicitly engage with transgender and/or non-binary embodied inequalities.

## Conclusions

The contribution of this paper is that it demonstrates how the research participants experience public space and how this impacts their active engagement with indoor water-based physical activity participation. There is evidence of high levels of public surveillance of transgender and non-binary people and this means scales of safety that are negotiated on a daily basis, including making decisions about physical activity. Feelings of being safe and/or unsafe to take part in physical activity extend to embodiment and display of the transgender and non-binary body. Combined, these elements affect opportunity to go swimming at an indoor public pool. It is evident that space and embodiment give rise to intricate exclusions. In this way, they are key components of inequality for transgender and non-binary individuals and communities. As such, they should be considered when devising initiatives, policy, and practice for transgender and non-binary inclusion, especially in swimming.

For the research participants swimming is an activity that has many positive outcomes, as long as it is within the group's exclusive use of a public pool: “It's really nice to be with [group name] in such a safe way … with swimming it's exercise, you also have fun and you mingle with people in the water” (Ean, one-to-one interview, 10th May 2019). Blue space is known to have a number of health-related benefits and yet there is limited social science research of indoor swimming pool watery space and immersion, play and recreational swimming by marginalized groups. For the research participants embodied gender dysphoria related to clothing and stages of surgery was shown to reduce when people were in the water. Given sport and physical activity are embodied-focused, the lack of knowledge of transgender and non-binary bodies and swimming potentially affects efforts to increase inclusion and reduce inequality.

This research project was limited to a local, established social group of transgender and non-binary individuals. The group consisted of, almost entirely, ethnic white people. Similar swim groups are apparent in urban areas elsewhere in the UK and more could be achieved through a national project that documents transgender and non-binary ethnic inequalities. Additionally, future research on physical activity participation can focus on the value of self-identification by participants and how stakeholders might respond to self-defined embodiments of sex and gender, especially in swimming contexts.

The long-term intent following the final stages of the local project, which involved introducing a pay-as-you-go once-a-month swim session for transgender and non-binary individuals, was to showcase the benefits of exclusive use of the indoor pool. The local stakeholder, the researcher and the transgender and non-binary social group supported the plan. However, the coronavirus global pandemic meant that the facility closed and the initiative ended abruptly. This was at a time when the project was shifting toward being sustainable and independent of funding. The closure limited the potential for transgender and non-binary advocacy to national sport governing bodies and national indoor pool providers. As with many research projects related to physical activity and inequality, Covid-19 has meant cessation, disruption and delay.

## Data Availability Statement

The raw data supporting the conclusions of this article will be made available by the authors, without undue reservation.

## Ethics Statement

The studies involving human participants were reviewed and approved by Bournemouth University. The patients/participants provided their written informed consent to participate in this study.

## Author Contributions

The author confirms being the sole contributor of this work and has approved it for publication.

## Conflict of Interest

The author declares that the research was conducted in the absence of any commercial or financial relationships that could be construed as a potential conflict of interest.

## Footnotes

^1^Cisgender is defined by Stonewall as “someone whose gender identity is the same as the sex they were assigned at birth. Non-trans is also used by some people” (https://www.stonewall.org.uk/help-advice/faqs-and-glossary/glossary-terms). The term can be abbreviate to “cis” or cis people.
